# Ventilatory compensation during the incremental exercise test is inversely correlated with air trapping in COPD

**DOI:** 10.12688/f1000research.20444.2

**Published:** 2020-03-10

**Authors:** Rottem Kuint, Neville Berkman, Samir Nusair

**Affiliations:** 1 Institute of Pulmonary Medicine, Hadassah-Hebrew University Medical Center, Jerusalem, Israel; 2 Rokach Center for the Prevention of Lung Diseases, Clalit Health Services, Jerusalem Region, Affiliated to The School of Medicine, Hebrew University and Hadassah, Jerusalem, Israel

**Keywords:** air trapping, COPD, exercise, isocapnic buffering phase, ventilatory equivalent

## Abstract

**Background:** Air trapping and gas exchange abnormalities are major causes of exercise limitation in chronic obstructive pulmonary disease (COPD). During incremental cardiopulmonary exercise testing, actual nadir values of ventilatory equivalents for carbon dioxide (V _E_/VCO _2_) and oxygen (V _E_/VO _2_) may be difficult to identify in COPD patients because of limited ventilatory compensation capacity. Therefore, we aimed in this exploratory study to detect a possible correlation between the magnitude of ventilation augmentation, as manifested by increments in ventilatory equivalents from nadir to peak exercise values and air trapping, detected with static testing.

**Methods:** In this observational study, we studied data obtained previously from 20 COPD patients who, during routine follow-up, underwent a symptom-limited incremental exercise test and in whom a plethysmography was obtained concurrently. Air trapping at rest was assessed by measurement of the residual volume (RV) to total lung capacity (TLC) ratio (RV/TLC). Gas exchange data collected during the symptom-limited incremental cardiopulmonary exercise test allowed determination of the nadir and peak exercise values of V _E_/VCO _2_ and V _E_/VO _2_, thus enabling calculation of the difference between peak exrcise value and nadir values of  V _E_/VCO _2_ and V _E_/VO _2_, designated ΔV _E_/VCO _2_ and ΔV _E_/VO _2_, respectively.

**Results:** We found a statistically significant inverse correlation between both ΔV _E_/VCO _2_ (r = -0. 5058, 95% CI -0.7750 to -0.08149, p = 0.0234) and ΔV _E_/VO _2_ (r = -0.5588, 95% CI -0.8029 to -0.1545, p = 0.0104) and the degree of air trapping (RV/TLC). There was no correlation between ΔV _E_/VCO _2_ and forced expiratory volume in the first second, or body mass index.

**Conclusions:** The ventilatory equivalents increment to compensate for acidosis during incremental exercise testing was inversely correlated with air trapping (RV/TLC).

## Abbreviations

BMI, body mass index; COPD, chronic obstructive pulmonary disease; FEV _1_, forced expiratory volume in the first second; GOLD, global initiative for chronic obstructive lung disease; IC, inspiratory capacity; RV, residual volume; TLC, total lung capacity; VCO _2_, CO _2_ output; V _E_, minute ventilation; V _E_/VCO _2_, ventilatory equivalent for CO _2_; V _E_/VO _2_, ventilatory equivalent for oxygen; ΔV _E_/VCO _2_, nadir to peak value of V _E_/VCO _2_; ΔV _E_/VO _2_, nadir to peak value of V _E_/VO _2_; VO _2_, oxygen consumption; Vt/VC, tidal volume to vital capacity ratio

## Introduction

Chronic obstructive pulmonary disease (COPD) patients often demonstrate significant exercise limitation, chiefly resulting from gas exchange abnormalities and ventilation-perfusion mismatching ^1^. This situation is often compounded by hyperinflation and air trapping with dynamic hyperinflation during exercise and gradual reduction of inspiratory capacity ^2^. To evaluate exercise capacity and determine the degree of exercise limitation and its mechanisms, the incremental exercise test is often applied, during which several ventilatory events occur and draw close attention.

During incremental cardiopulmonary exercise testing, the ratio of minute ventilation (V _E_) to carbon dioxide output (VCO _2_) and to oxygen consumption (VO _2_), also known as ventilatory equivalent for carbon dioxide (V _E_/VCO _2_) and oxygen (V _E_/VO _2_), respectively, serve to evaluate ventilatory efficiency. The ventilatory threshold, a term coined in the context of gas exchange measurements and represents to a great extent the VO _2_ at anaerobic threshold, heralds the onset of the isocapnic buffering phase, in which there is an increased contribution of anaerobic metabolism to provide the energy required for the increasing demands of exercise, accompanied by increased CO _2_ production with corresponding ventilation increase. With the accumulation of lactic acid and H ^+^ protons, as the bicarbonate reserves are decreased beyond the isocapnic buffering phase, an augmented ventilatory response starts at the ventilatory compensation point, which is disproportionate to the degree of CO _2_ production, leading to V _E_/VCO _2_ surge towards peak exercise, a phenomenon termed the ventilatory compensation phase.

The V _E_/VCO _2_ is often increased as a result of ventilation-perfusion mismatching ^1, 3^. The nadir V _E_/VCO _2_ value during incremental exercise is inversely related to the degree of exercise limitation ^4^, as well as the severity of airway obstruction, as determined by the values of forced expiratory volume in first second (FEV _1_) ^5, 6^. The nadir value is considered to reflect ventilatory efficiency as higher values obtained at the early stages of the incremental exercise test result from frequently encountered hyperventilation. Interestingly, marked hyperinflation and reduced ventilatory capacity tend to reduce ventilatory equivalents as ventilation may become constrained. Both baseline and peak exercise V _E_/VCO _2_ values were found to be lower in patients with more severe airway obstruction as V _E_ is decreased relative to VCO _2_ in these patients ^5, 6^.

Although COPD patients may terminate exercising at an early stage during the incremental exercise test due to airflow obstruction and air trapping, suboptimal effort and deconditioning, the latter of which may be associated with somewhat earlier development of metabolic acidosis, may also lead to early termination of the exercise test. Regardless of the reason causing decreased exercise performance, the ventilatory compensation phase may not be demonstrated. Ventilatory equivalents during incremental exercise may be affected by the above-mentioned processes in different directions. In this exploratory study we sought to confirm the presumption that the ability to achieve ventilatory compensation in response to acidosis is related to the degree of air trapping, with the hypothesis that the ability to augment ventilation beyond the ventilatory compensation point in COPD patients is inversely correlated with the degree of air trapping.

## Methods

### Study design and subjects

A retrospective analysis of data obtained from the medical records of COPD patients who underwent incremental cardiopulmonary exercise testing in the pulmonary function laboratory of the Institute of Pulmonary Medicine in the Hadassah Medical Center, Jerusalem, Israel between June 2010 and August 2016, and in whom whole body plethysmography was performed concurrently, as part of their routine clinical evaluation. The number of patients was determined by the availability of incremental exercise test and plethysmography data that was performed concurrently. Subjects were previously diagnosed with COPD based on post-bronchodilator spirometry showing an FEV _1_ to forced vital capacity (FVC) ratio (FEV _1_/FVC) of ≤0.7, regardless of the value of FEV _1_ (according to 2017 GOLD guidelines). Comorbidities such as obesity, cardiac disease and use of beta blockers were noted, but were not a cause to exclude patients from the study. The research ethics board (REB) of the Hadassah Medical Organization (protocol No. 0040-16-HMO) approved data collection and waived the need for informed consent, as this research was retrospective, did not affect patient management, or involve collecting biospecimens.

### Procedures

Pulmonary function tests and exercise tests were performed by technicians in the presence of a physician during exercise tests. Whole-body plethysmography was performed using a commercially available body plethysmograph (Elite series, MedGraphics). Spirometry performance and slow vital capacity determination were followed by assessment of lung volumes, which was performed by direct measurement of thoracic gas volume (TGV), from which the residual volume (RV), total lung capacity (TLC) and RV/TLC ratios could be calculated as the primary method to estimate gas trapping. Additionally, we determined the ratio of inspiratory capacity (IC) to total lung capacity, IC/TLC, which has been proposed to reflect lung expansion as a result of reduced lung recoil in emphysema ^7^. Using the same lung function system, transfer factor of the lung for carbon monoxide (TL _CO_) was measured by the single breath method.

Patients performed incremental symptom-limited cycle ergometry connected to a metabolic system with cycle ergometer (Ultima CardiO _2_, MedGraphics). For each patient, baseline measurements were obtained during the resting period of 1 minute, after which the patient would start cycling at a constant rate of 50–60 rpm. Following a 1–2 minute warm-up period of unloaded exercise and with VO _2_ and VCO _2_ reaching a plateau, a ramp protocol was started with a workload increase of 15–25 watt per minute, depending on the predicted maximal VO _2_ and the general state of the patient. The test was terminated at exhaustion, when the patient could not keep the required cycling pace or asked for exercise termination. Routine precordial 12-lead electrocardiogram monitoring, continuous measurements of V _E_, VO _2_, VCO _2_ (averaged every 10 seconds), heart rate and finger arterial pulse oximetry were recorded. The peak VO _2_ and the ventilatory threshold were both noted, as well as nadir ventilatory equivalents (V _E_/VCO _2_ and V _E_/VO _2_). Values of V _E_/VCO _2_ and V _E_/VO _2_ obtained at the termination of loaded exercise (peak exercise) enabled calculation of the difference between peak exercise value and nadir values of V _E_/VCO _2_ and V _E_/VO _2_ (designated ΔV _E_/VCO _2_ and ΔV _E_/VO _2_, respectively).

### Statistical analysis

Statistical analysis was performed using the GraphPad Prism 3.0 program software and Spearman’s test was used for correlation analysis, with calculation of the Pearson correlation coefficient (r) between measured parameters. Correlations were considered of statistical importance if two-tailed p value was <0.05. Unpaired t-test was used to compare between groups, and a two-tailed p value < 0.05 was considered statistically significant

### Standards of reporting

This manuscript is compliant with the STROBE guideline.

## Results

In total, 20 patients were included in our analysis with a mean ± SD age of 63 ± 10 years (range 37–77). None of the patients had cardiac failure. Two patients were receiving beta blockers. Table 1 summarizes clinical data of these patients.

**Table 1.** Patient clinical, pulmonary function and incremental exercise test data.

**Age (years)**	63 ± 10
**Gender (% of males)**	80
**BMI (kg/m ^2^)**	27.2 ± 6.8
**Static pulmonary function tests**	
FEV _1_ (L)	1.80 ± 0.70
FEV _1_ (% predicted)	63 ± 21
FVC (L)	3.07 ± 1.03
FVC (% predicted)	85 ± 22
FEV _1_/FVC (%)	59 ± 12
RV (L)	3.82 ± 1.82
RV (% predicted)	166 ± 60
IC (L)	2.1 ± 0.6
IC (% predicted)	73 ± 21
TLC (L)	6.94 ± 1.81
TLC (% predicted)	111 ± 24
RV/TLC (%)	55 ± 11
IC/TLC (%)	31 ± 8
TL _CO_ (% predicted)	67 ± 17
**No. of patients according to GOLD** **classification (% of all patients)**	
GOLD I	7 (35%)
GOLD II	8 (40%)
GOLD III	4 (20%)
GOLD IV	1 (5%)
**Exercise test**	
Peak Work Rate (watt)	94 ± 30
Peak VO _2_ (ml/kg/min)	16.4 ± 4.7
Peak VO _2_ (% predicted maximal VO _2_)	69 ± 14
Peak RER	1.13 ± 0.13
VE/VCO _2_ slope	34 ± 6
Nadir V _E_/VCO _2_	36 ± 7
Peak exercise V _E_/VCO _2_	39 ± 8
ΔV _E_/VCO _2_	2.6 ± 3.4
Nadir V _E_/VO _2_	37 ± 7
Peak exercise V _E_/VO _2_	45 ± 10
ΔV _E_/VO _2_	8.0 ± 7.6
Peak heart rate (% predicted maximal heart rate)	80 ± 11
Breathing reserve * (%)	30 ± 15
MVV-V _E_ (L/min)	25.9 ± 19.1
Vt/VC at peak exercise (%)	46 ± 8
Arterial blood O _2_ desaturation at peak exercise (%)	2.4 ± 3.6
Resting P _ET_CO _2_ (mmHg)	31.8 ± 5.7
Peak exercise P _ET_CO _2_ (mmHg)	33.9 ± 7.4

Values are mean ± SD. BMI, body mass index; FEV _1_, forced expiratory volume; FVC, forced vital capacity; HR, heart rate; GOLD, Global initiative for chronic Obstructive Lung Disease; IC, inspiratory capacity; MVV-VE, difference between maximal voluntary ventilation and peak ventilation; P _ET_CO _2_, end-tidal PCO _2_; RER, respiratory exchange ratio; RV, residual volume; TLC, total lung capacity; TL _CO_, transfer factor of the lung for carbon monoxide; VO _2_, oxygen consumption; V _E_/VO _2_, ventilatory equivalent for oxygen; V _E_/VCO _2_, ventilatory equivalent for carbon dioxide; Δ is the difference between measured value of ventilatory equivalent at peak exercise and nadir value ; Vt/VC, tidal volume to vital capacity ratio.

*Breathing reserve = (maximal voluntary ventilation - peak ventilation) / maximal voluntary ventilation.

We found a statistically significant inverse correlation between both ΔV _E_/VCO _2_ (r = -0.5058, 95% CI -0.7750 to -0.08149, p = 0.0234) and ΔV _E_/VO _2_ (r = -0.5588, 95% CI -0.8029 to -0.1545, p = 0.0104), with the degree of air trapping as estimated by the RV/TLC ratio ( Table 2). In this cohort, there was a significant correlation between Nadir VE/VCO2 and VE/VCO2 slope (r=0.82, 95% CI 0.59 to 0.92, p<0.0001).In contrast to increments in ventilatory equivalents, there was no correlation between the nadir values of the ventilatory equivalents and RV/TLC ( Table 2). Interestingly, inspiratory capacity to total lung capacity ratio (IC/TLC), which reflects pulmonary expansion in emphysema, did not show a correlation with either ΔV _E_/VCO _2_ or ΔV _E_/VO _2_ ( Table 2).

**Table 2.** Statistical correlation analysis of nadir to peak increment (Δ) ventilatory equivalents or nadir ventilatory equivalents and parameters of static pulmonary function, incremental exercise and BMI.

	R	95% CI for r	p value
ΔV _E_/VCO _2_ to RV/TLC	-0.5059	-0.7750 to -0.0815	0.0229
ΔV _E_/VO _2_ to RV/TLC	-0.5589	-0.8029 to -0.1545	0.0104
V _E_/VCO _2_ slope to RV/TLC	-0.2702	-0.6367 to 0.1958	0.2493
V _E_ intercept to RV/TLC	0.3542	-0.1049 to 0.6888	0.1255
ΔV _E_/VCO _2_ to IC/TLC	0.2835	-0.1819 to 0.6452	0.2258
ΔV _E_/VO _2_ to IC/TLC	0.2369	-0.2298 to 0.6150	0.3145
ΔV _E_/VCO _2_ to FEV _1_	0.2902	-0.1749 to 0.6494	0.2145
Nadir V _E_/VCO _2_ to FEV _1_	0.0894	-0.3677 to 0.5118	0.7077
ΔV _E_/VCO _2_ to peakVO _2_ (as %predicted VO _2_max)	0.0902	-0.3670 to 0.5124	0.7051
ΔV _E_/VCO _2_ to peakVO _2_ (in ml/kg/min)	0.4506	0.0100 to 0.7447	0.0462
ΔV _E_/VCO _2_ to TL _CO_	0.0015	-0.4531 to 0.4555	0.9951
Nadir V _E_/VCO _2_ to TL _CO_	-0.5281	-0.7923 to -0.0971	0.0201
Nadir V _E_/VCO _2_ to RV/TLC	-0.1316	-0.5426 to 0.3303	0.5803
Nadir V _E_/VO _2_ to RV/TLC	-0.0640	-0.4927 to 0.3896	0.7885
ΔV _E_/VCO _2_ to BMI	-0.3809	-0.7047 to 0.0742	0.0975
ΔV _E_/VO _2_ to BMI	-0.3399	-0.6802 to 0.1210	0.1426

p value <0.05 is considered of statistical importance. r, Pearson correlation coefficient; BMI, body mass index; IC, inspiratory capacity; RV, residual volume; TL _C_, total lung capacity; FEV _1_, forced expiratory volume; TL _CO_, transfer factor of the lung for carbon monoxide; VO _2_, oxygen consumption; V _E_/VCO _2_, ventilatory equivalent for carbon dioxide; V _E_/VO _2_, ventilatory equivalent for oxygen; Δ is the difference between measured value at peak exercise and nadir value

We examined possible correlation between ΔV _E_/VCO _2_ and other static parameters of pulmonary function or findings on exercise testing (summarized in Table 2). Notably, we found no significant correlation between neither ΔV _E_/VCO _2_ nor nadir V _E_/VCO _2_ and peak VO _2_ (% of predicted VO _2_max) or FEV _1_ (% of predicted). There was a correlation between ΔV _E_/VCO _2_ and peakVO _2_ (in ml/kg/min) which just achieved a significance at p= 0.0462, ( Table 2). As expected ^1^, nadir values of V _E_/VCO _2_ correlated with TL _CO_ (r = -0.5281, 95% CI -0.7923 to -0.09708, p = 0.0201); however, we did not find a correlation between ΔV _E_/VCO _2_ and TL _CO_ ( Table 2).

In an attempt to identify factors related to the lack of increment of V _E_/VCO _2_ besides those related to air trapping, we compared values of parameters related to static pulmonary functions and those related to performance of exercise in patients with and without increment in V _E_/VCO _2_ (i.e., ΔV _E_/VCO _2_ = 0) ( Table 3). We noted a statistically important difference in peak RER, VO2 (in ml/kg/min), peak WR; However, FEV _1_, IC/TLC, TL _CO_, peak VO _2_ (as % of predicted VO _2_), and nadir V _E_/VCO _2_ were not different between these two groups. RV/TLC had a statistically important difference between the two groups. However, RV/TLC as well as peak VO _2_ adjusted to body weight are inherently derived from the parameters that the correlation study had examined and therefore, this difference is not surprising. There was also a significant difference in the values of peak exercise end-tidal PCO _2_ between the two groups with lower values of end-tidal PCO _2_ in patients who had ΔV _E_/VCO _2_>0. Interestingly, patients who did not have a V _E_/VCO _2_ increment at peak exercise, had a trend towards a higher peak exercise end-tidal PCO _2_ than their baseline values (37.3±8.1 vs 32.5±6.7) although the difference did not achieve a statistically important difference (p=0.1651). Moreover, the value of V _E_/VCO _2_ y-abcissa intercept (i.e., V _E_ intercept) in patients who did not have an increment of V _E_/VCO _2_ (i.e., ΔV _E_/VCO _2_=0) was significantly higher than in those who had a ΔV _E_/VCO _2_>0. Variables that could be affected by ventilatory mechanical constraints during exercise, the breathing reserve and peak tidal volume to vital capacity ratio (Vt/VC) were not different between the two groups.

**Table 3.** Comparison between patients in whom there was no nadir to peak exercise increment in V _E_/VCO _2_ (ΔV _E_/VCO _2_=0) and patients with nadir to peak exercise increment in ΔV _E_/VCO _2_ (ΔV _E_/VCO _2_>0).

	ΔV _E_/VCO _2_=0 (n=10)	ΔV _E_/VCO _2_>0 (n=10)	p
RV/TLC (%)	60.3 ± 7.7	49.4 ± 11.1	0.0200
peak RER	1.05 ± 0.13	1.20 ± 0.10	0.0114
Peak Work Rate (Watt)	81 ± 30	108 ± 25	0.0382
Nadir V _E_/VCO _2_	35.9 ± 8.3	37.2 ± 5.5	0.6849
V _E_/VCO _2_ slope	32 ± 7	35 ± 5	0.3297
V _E_ intercept (L/min)	6.08 ± 2.29	3.28 ± 2.61	0.0201
VO _2_peak (% predicted maximal VO _2_)	67.9 ± 18.4	71.3 ± 8.0	0.5992
VO _2_peak (ml/kg/min)	14.3 ± 4.7	18.5 ± 3.8	0.0411
Oxygen Pulse (ml/min/beat)	9.1 ± 3.2	9.8 ± 1.4	0.5318
Baseline end-tidal PCO _2_ (mmHg)	32.5 ± 6.7	31.2 ± 4.8	0.6230
Peak exercise end-tidal PCO _2_ (mmHg)	37.3 ± 8.1	30.5 ± 4.8	0.0350
FEV _1_ (% predicted)	55.9 ± 21.2	70.5 ± 18.9	0.1214
FEV _1_/FVC	0.56 ± 0.14	0.62 ± 0.09	0.2713
Breathing reserve * (%)	31 ± 17	28 ± 14	0.7166
Vt/VC at peak exercise (%)	45 ± 9	48 ± 6	0.3518
IC/TLC (%)	29.2 ± 8.5	32.9 ± 7.1	0.3005
TL _CO_ (%predicted)	70.4 ± 19.8	63.7 ± 14.3	0.4023
BMI (Kg/m ^2^)	29.3 ± 7.7	25.1 ± 5.4	0.1823

Values are mean ± SD. p<0.05 signifies an important difference. RER, respiratory exchange ratio; RV, residual volume; TLC, total lung capacity; TL _CO_, transfer factor of the lung for carbon monoxide; VO _2_, oxygen consumption; FEV _1_, forced expiratory volume; IC, inspiratory capacity; V _E_/VCO _2_, ventilatory equivalent for CO _2_; ΔV _E_/VCO _2_ is the difference between measured value at peak exercise and nadir value of V _E_/VCO _2_; Vt/VC, tidal volume to vital capacity ratio *Breathing reserve = (maximal voluntary ventilation - peak ventilation) / maximal voluntary ventilation.

## Discussion

Inefficient ventilation in patients with COPD and particularly emphysema is often evidenced by elevated nadir V _E_/VCO _2_. The elevation of nadir V _E_/VCO _2_ may be related to the extent and severity of emphysematous changes in the lungs. It has been shown that in patients with mild to moderate airflow decrease, the values of nadir V _E_/VCO _2_ correlated with the percentage of low attenuation areas on computerized tomography ^8, 9^ as well as the decrease in pulmonary perfusion, as estimated by the inert gas rebreathing method ^9^. Interestingly, in smokers without COPD, the degree of CO diffusion decrease correlated with elevation of nadir V _E_/VCO _2_ during the incremental exercise test ^10^, again pointing to a strong relation between impaired ventilation efficiency and gas exchange ability. Notably, significantly increased ventilatory constraints, as occurs in marked air trapping, decrease V _E_, thus leading to lower nadir V _E_/VCO _2_ in more severe airway obstruction ^2, 6^.

In this study we examined the ability of COPD patients during incremental exercise testing to increase ventilation towards peak exercise, focusing on the increase in V _E_/VCO _2_ and V _E_/VO _2_ that occur following the ventilatory compensation point. We quantified this phenomenon measuring the difference between the values of V _E_/VCO _2_ and V _E_/VO _2_ at nadir and peak exercise obtained post the ventilatory compensation point, which we termed ΔV _E_/VCO _2_ and ΔV _E_/VO _2_, respectively. We found that the ability to increase ventilation during incremental exercise testing in response to metabolic acidosis, as normally occurs in higher exercise intensities beyond the ventilatory compensation point, was diminished in COPD patients and in correlation with the severity of air trapping as represented by RV/TLC obtained at rest. Furthermore, this may implicate a difficulty in coping with the ventilatory demands of metabolic acidosis resulting from states such as acute renal failure or septic shock, thus ΔV _E_/VCO _2_ (and seemingly ΔV _E_/VO _2_) may allow assessment of the ability of COPD patients to withstand such metabolic challenges. Limited ventilatory compensation is clinically significant as it has been shown that people with COPD have higher arterial PCO _2_ and lower pH in response to blood lactate elevation during exercise, compared to people without COPD ^11^. In our cohort, peak end-tidal PCO _2_ in patients without V _E_/VCO _2_ increment had a non-significant trend towards higher values than baseline end-tidal PCO _2_ thus pointing to a limited ability to compensate for acidosis. Our findings also show that patients who did not have an increment in V _E_/VCO _2_ had higher average V _E_ intercept value of the V _E_/VCO _2_ slope than patients with ΔV _E_/VCO _2_>0, thus suggesting a role for the presence of higher dead space breathing ^5^ in patients who do not increase ventilatory equivalents and produce ventilatory compensation.

One way in which pulmonary hyperinflation can be expressed is by the increased ratio of RV/TLC, which reflects air trapping and is associated with airway narrowing. Alternatively, IC/TLC may also be decreased because of lung expansion associated with emphysematous changes and reduced lung recoil. A recent study showed that either increased RV/TLC or decreased IC/TLC or both simultaneously can be present, with airway narrowing on imaging being associated with increased RV/TLC and emphysema associated with deceased IC/TLC ^7^. Therefore, it seems that the lack of increments in ventilatory equivalents in our study is correlated with air trapping but not to the mere presence of emphysema.

One may argue that the limited rise of ventilatory equivalents beyond the ventilatory threshold during the incremental exercise test may reflect sub-optimal exercise performance; however, we think that the lower respiratory exchange ratio at peak exercise may represent another manifestation of the decreased ventilatory response.

Although both ΔV _E_/VO _2_ and ΔV _E_/VCO _2_ seem to show similar correlation to RV/TLC and utility in estimating the ventilation response to acidosis under these conditions, a further study with larger numbers of patients may help distinguish the different roles of these parameters.

This study has several limitations. The retrospective design and the small number of patients included are important limitations, in particular when comparing two subgroups. However, these limitations may be of lesser significance when searching for correlations. Measured increment of ventilatory equivalents in a significant number of patients was nil, with a value =0. This may point to an important limitation of this measurement. Future studies should be designed to utilize a lower ramp than the ramps that were used in our patients studied retrospectively, to allow time for patients to adjust and generate ventilatory compensation, something that may be more difficult to do exercising at high work rates. Some patients were obese, which may have caused mild restriction, somewhat contributing to air trapping. However, we didn’t find a correlation between body mass index (BMI) and either ΔV _E_/VCO _2_ or ΔV _E_/VO _2_. Unfortunately, dynamic hyperinflation, a well-known phenomenon that is associated with ventilatory constraints during exercise in COPD, was not assessed by performing repeated inspiratory capacity maneuvers during exercise and therefore, we cannot describe the relation between dynamic hyperinflation (i.e. changes in IC/TLC) and ventilatory equivalents increment in this cohort. However, dynamic hyperinflation developing during exercise is associated with changes in Vt/VC at peak exercise with lower value than the expected portion of 50–60% at peak incremental exercise ^12^ as dynamic hyperinflation develops with increasing work rate during exercise performance ^2^. The average Vt/VC at peak exercise of all patients in our cohort was 46±8%, without a statistically meaningful difference in peak exercise Vt/VC between those who had V _E_/VCO _2_ increment and those who didn’t ( Table 3). Therefore, it is unlikely that development of dynamic hyperinflation can differentiate between ventilatory equivalent increment patterns during exercise in our patients.

In conclusion, ventilation augmentation during incremental exercise testing at exercise intensities beyond the ventilatory compensation point in COPD patients was diminished in correlation with the severity of air trapping. Future studies will confirm the clinical usefulness of measuring ΔV _E_/VCO _2_ and ΔV _E_/VO _2_, including their potential role as serially measured physiologic parameters to assess effects of interventions (e.g. pulmonary rehabilitation) in COPD.

## Data availability

### Underlying data

Harvard Dataverse: Ventilatory Equivalents increment during Exercie in COPD. https://doi.org/10.7910/DVN/QTCV0M ^13^.

This project contains the following underlying data:

- nadir to peak VE-VCO2 during exercise in COPD.tab (demographic information and parameter measurements for each patient).

Data are available under the terms of the Creative Commons Zero “No rights reserved” data waiver (CC0 1.0 Public domain dedication).
