# Supplementary material for: Ventilatory compensation during the incremental exercise test is inversely correlated with air trapping in COPD
Source: F1000Res. 2020 Mar 10;8:1661. Originally published 2019 Sep 19. [Version 2] doi: 10.12688/f1000research.20444.2 (PMC7122427; doi:10.12688/f1000research.20444.2)
Supplement: Peer review history

# Peer review: Reviewer response for version 2

**DOI:** 10.5256/f1000research.24678.r61120

**Published:** 2020-04-01

**Authors:** Gabriele Valli

**Affiliations:** 1 Emergency Department, San Giovanni Addolorata Hospital, Rome, Italy

The new version has been improved and I have no further comments.

I confirm that I have read this submission and believe that I have an appropriate level of expertise to confirm that it is of an acceptable scientific standard.

# Peer review: Reviewer response for version 2

**DOI:** 10.5256/f1000research.24678.r61119

**Published:** 2020-03-10

**Authors:** William W. Stringer

**Affiliations:** 1 Lundquist Institute for Biomedical Innovation at Harbor-UCLA Medical Center, Torrance, CA, USA

The revised version is acceptable for indexing.

I confirm that I have read this submission and believe that I have an appropriate level of expertise to confirm that it is of an acceptable scientific standard.

# Peer review: Reviewer response for version 1

**DOI:** 10.5256/f1000research.22475.r57517

**Published:** 2019-12-09

**Authors:** William W. Stringer

**Affiliations:** 1 Lundquist Institute for Biomedical Innovation at Harbor-UCLA Medical Center, Torrance, CA, USA

Summary

                 Drs Kuint, Berkman, and Nusair retrospectively analyzed 20 COPD patients (GOLD 1: #7, II: #8, III: #4, IV: #1) who had full PFTs with lung volumes and a CPET test over a 7 year period (2010 to 2016). They were interested in air trapping and the effect on exercise performance, specifically trying to better understand the changes in Ventilatory Equivalents for Oxygen and Carbon Dioxide during incremental exercise tests on a cycle ergometer (nadir value to peak value). Further, they relate these changes to resting pulmonary function testing. The study was retrospective. They conclude that “ventilatory equivalents increment to compensate for acidosis during incremental exercise testing was inversely correlated with air trapping (RV/TLC) and may be a candidate prognostic biomarker.” The study is well written.

 Major Issues

1. The study would have been greatly assisted by obtaining inspiratory capacity (IC maneuvers) during exercise to document dynamic hyperinflation. 
2. The study is retrospective, is a very small group, and was collected across 7 years (2010-2016, about 2-3 subjects per year), meaning that there is great potential for changes in devices, calibration, technician training, etc. that may have affected the PFT and CPET measurements.
3. The GOLD III and IV subjects are very poorly represented (Gold III (20%) and Gold IV (5%)), i.e. most subjects (75% were Gold I or II). Per the Neder and Thirapatarapong references in your paper, Gold I-II have lower ETCO2 and higher VE/VCO2 reflecting less efficient ventilation, and GOLD III-IV have higher ETCO2 and lower VE/VCO2 reflecting mechanical limitation.
4. This paper is also hampered by so many of the changes in VE/VCO2 (10) and VE/VO2 (7) from peak-nadir were nil, i.e. = "0"
5. There is no analysis of VE/VCO2 slope or intercept reported in the paper or data spreadsheet. 
6. No blood gases (to document acidosis or lactate levels) or end tidal CO2 values to document changes in ventilatory efficiency. Importantly, there is no discussion of the determinants of VE/VCO2, i.e. VE/VCO2 i.e., =  k /( PaCO2) * ( 1- VD/VT) and how these two variables interact in the current study.
7. COPD patients are less likely to protect their arterial pH and arterial CO2 during exercise (Casaburi, et al, Am Rev Respir Disease, 143: 9-18, 1991 ^1^)  See figure # 8.  There is no discussion of this important feature of COPD patients in the manuscript.

Minor Issues

1. Introduction, page 3, First Paragraph, line 2. “Effort” should likely be exercise or activity.
2. Introduction, page 3, First Paragraph, line 3+4. Here you mention gas exchange abnormalities and ventilation perfusion mismatching, but in paragraph 4, lines 3 you also discuss deconditioning. All three mechanisms appear to be important and should be mentioned in both places.
3. Introduction, page 3, Second Paragraph, line 14.  “exhausted” should be “decreasing”.
4. Introduction, page 3, Third Paragraph, line 6 and 14. Reference 5 and 6 would both seem to contribute to the concepts here, and should both be quoted. 
5. Methods, Paragraph 1, Study Design and Subjects, lines 1-5.  How are the authors confident that all subjects with COPD and full PFTs studied in the laboratory were identified for the current manuscript?
6. Methods, Paragraph 1+2, Procedures. Were there any changes in PFT or CPET equipment, calibration, or technician protocols during the 7 years of data collection that might affect spirometry, lung volumes, gas transfer or CPET variables? 
7. Methods, Paragraph 2, Procedures. 15-25 watts appears to be a very large ramp increment in patients with moderate to severe COPD. Please explain why these very large numbers were chosen, and how that may have affected the findings in the study (i.e. short ramp times may decrease time for ventilatory compensation of metabolic acidosis).
8. Table 1 – Consider including peak VO2/kg/kg/min, peak WR, peak RER, MMVV-VE (L/min), and end tidal CO2 values for the various subjects (and in the accompanying spreadsheet). **The spreadsheet accompanying the article is very helpful and the authors should be commended for making the data available.  **A large number of subjects (~ 70%) had a VE/MVV below 85% or > 15 liters/minute (MVV-VE) at end exercise, indicating a lack of ventilatory limitation in most of these subjects.
9. Figure 1 – As mentioned above, a large number of the subjects had no change in DVE/VCO2 or DVE/VO2. Although Table 3 addresses it, it would appear that the authors have neglected to mention this as a limitation of the entire study in the discussion.

I confirm that I have read this submission and believe that I have an appropriate level of expertise to state that I do not consider it to be of an acceptable scientific standard, for reasons outlined above.

## References

[1] : Reductions in exercise lactic acidosis and ventilation as a result of exercise training in patients with obstructive lung disease. *Am Rev Respir Dis*.1991;143(1) : 10.1164/ajrccm/143.1.9 9-18 10.1164/ajrccm/143.1.9 1986689

# Response

**Published:** 2020-03-04

**Authors:** Samir Nusair

**Affiliations:** Clalit Health Services, Israel

We would like to thank Prof. Stringer for his valuable comments. This a retrospective study with small number of patients, and we certainly acknowledge the inherent significant limitations, which are referred to in the Discussion section We consider this work as a preliminary/exploratory study, and as that it has well acknowledged limitations and implications. We added the term “exploratory study” in the abstract and the introduction section to allude to that fact. 

   

 We would like to address the concerns raised by the Reviewer.

 Re Major comments:

 1. At the time of performance of these CPET studies, we did not routinely perform repeated inspiratory capacity maneuvers during exercise to allow detecting dynamic hyperinflation, because of lack of both software add-on and experience in performing theses maneuvers.

 Notably, dynamic hyperinflation is associated with changes in tidal volume/vital capacity ratio (Vt/VC) towards peak exercise with lower ratios of Vt/VC as dynamic hyperinflation is more evident during exercising at higher workloads.    

 (Ref: O'Donnell DE, Revill SM, Webb KA. Dynamic hyperinflation and exercise intolerance in chronic obstructive pulmonary disease. Am J Respir Crit Care Med. 2001;164:770-7)

 All in all there was a very little decrease in Vt/VC obtained at peak exercise, with average values of 46% +8% (Table 1).

 We now added to Table 3 a comparison of Vt/VC values of patients who had an increment in VE/VCO2 as opposed to patients who did not (deltaVE/VCO2=0). We found that the peak exercise Vt/VC did not differ between the two groups. Despite the limitations of this method, we conclude that dynamic hyperinflation probably did not play a key role in preventing VE/VCO2 increment from nadir values to peak exercise in our patients. The last paragraph in the Results section was altered to describe the added data and findings, and also description of findings investigating possible ventilatory mechanical constraints. 

 Additionally, the Discussion alluded to the possible use of Vt/VC as a way to evaluate mechanical constraints on ventilation (paragraph before last).  

 2. Throughout the years 2010-2016 we used the same CPET and plethysmography devices described in the methods and applied the same calibration methods as recommended by the manufacturer.  

 3. As patients have more severe obstructive abnormality (GOLD III-IV more than GOLD I-II), they have lower ventilation, with reduced VE/VCO2 and VE/VO2 values. This has been referred to in the Introduction section, paragraph 3. Certainly, if we had bigger number of patients and all GOLD classes of patients were represented equally, we may have been able to deduce important differences between these classes.  

 Data on end-tidal PCO2 has now been provided.

 4. The fact that many patients had no increment in ventilatory equivalents from nadir to peak exercise (i.e., deltaVE/VCO2=0) is a limitation of the measurement. As mentioned in the study limitations paragraph, this may as well be explained by suboptimal effort of the patients or the ventilatory limitations of these patients. We referred to this issue now in the Limitations paragraph.

 5. Data of VE/VCO2 slope was added. There was a significant correlation between Nadir VE/VCO2 and VE/VCO2 slope (R=0.82 95%CI 0.59-0.92, p<0.0001). Data of VE/VCO2 intercept was also added. (Represented in all 3 Tables).

 6. We do not take arterial blood gases during routine clinical CPET performance to keep the test acceptable for our patients. This may be considered in a future prospective study if approved by ethical board. Alternatively, there are minimally invasive methods to allow blood lactate measurements which we do not have available in our clinical service.  

 Regarding end-tidal CO2, we added the end-tidal PCO2 values at rest and at peak exercise. (See further, response to comment 7).

 7. The reviewer brings our attention to the findings from a previous study which shows that COPD patients have higher arterial PCO2 and lower pH in response to blood lactate elevation during exercise, compared to people without COPD.

 This is somewhat expected, as with the presence of ventilatory constraints, these patients are not able to develop sufficient ventilatory compensation during exercise.

 In this study we did not perform these measurements; However, end-tidal PCO2 did not decrease towards peak exercise, in the patients who did not have VE/VCO2 increments, thus probably in agreement with the previously published findings highlighted by the reviewer. We added a reference to the suggested paper in our discussion. Note that at the end of the discussion (paragraph 2), we refer to the possible implication of limited ability of COPD patients of coping with metabolic acidosis from other reasons (e.g., diabetic acidosis) when they are not able to generate ventilatory compensation.

 Re Minor Comments:

 1.  We agree with the reviewer and “effort” was changed to “exercise” as suggested.

 2. We agree with the reviewer regarding the importance of deconditioning as a reason for reduction in exercise capacity in COPD patients. However, the opening sentences were concentrated around describing the pulmonary mechanisms in exercise limitation in these patients. We do refer to deconditioning in the last paragraph of the Introduction.

 3. Introduction, paragraph 2:  “exhausted” was changed to “decreased” as suggested.

 4. Citations of References 5 & 6 were joined twice in the Introduction (Paragraph 3) as suggested.

 5. To identify patients and include them in the study, we looked into the CPET system software and identified all the patients that underwent CPET between the years 2010-2016 (on the same CPET device) and had a background diagnosis of COPD. Then from those patients we included only the patients that underwent body plethysmography concurrently in our laboratory, on the same day of CPET or within few days close to the date of CPET performance. 

 6. As stated above, there were no changes in the equipment during the 7 years.

 7. Ramp increment is routinely chosen to allow for predicted WR to be achieved within 8 minutes of exercise. Only one patient exercised at a ramp of 25W/min. All other patients had a ramp of 15-20W/min. We agree with the reviewer that lower ramps may be warranted for more debilitated patients, which we often offer our patients who report low activity level. However, most patients do not handle a longer period of loaded exercise, because of other factors such as claustrophobia and mental fatigue.

 Regarding the effect of short ramp times on ventilatory compensation, we agree with the reviewer that higher ramps may shorten the time interval of ventilatory compensation and make it harder to evaluate the ventilation increment during the respiratory compensation phase but we doubt that this might affect the findings of our study. Perhaps, a future prospective evaluation, with exercise tests performed at lower ramps may allow a better evaluation of ventilation increment towards peak exercise. A note on that is added in study limitations.

 8. We considered the suggestions of the Reviewer regarding the expanding of Table 1 to include data already shared with the readers in the accompanying research data file which is already available at data sharing site. Regarding peak VO2 in ml/min/kg, peak WR, peak RER, and MVV-VE, were added to Table 1 in the body of the article, and also in Table 3 which shows comparison between those who did not have VE/VCO2 increment and those who did. Interestingly, VO2 in ml/kg/min was found to be different between the two groups, unlike VO2 expressed as % of predicted VO2max.

 9. The absence of ventilatory equivalent increment (i.e., delta VE/VCO2=0) has now been addressed in the study limitations paragraph.

# Peer review: Reviewer response for version 1

**DOI:** 10.5256/f1000research.22475.r56449

**Published:** 2019-12-02

**Authors:** Gabriele Valli

**Affiliations:** 1 Emergency Department, San Giovanni Addolorata Hospital, Rome, Italy

In the present study, the authors investigate a possible correlation between the magnitude of ventilation augmentation (manifested by increments in ventilatory equivalents from nadir to peak effort) and the lung air trapping indexes measured at rest. The authors analyzed retrospectively data from 20 symptom-limited incremental exercise test performed by 20 COPD patients. Patients underwent to CPET as part of a routine evaluation. The differences between nadir to peak exercise of VE/CO _2_ were correlated to air trapping parameters measured at rest in a plethysmography. A positive correlation was found between nadir to peak exercise VE/CO _2_ and VE/VO _2_ and the degree of air trapping expressed as RV/TLC. The authors concluded that ventilatory equivalents increment (above anaerobic threshold) could be inversely correlated with air trapping and it may be a candidate prognostic biomarker.

 Although the paper is well written I have some comments to do:

1. The authors choose to calculate ventilatory equivalent subtracting the values at peak exercise to the values measured at the nadir, assuming that the reduced increment of ventilatory equivalent after the minimum values could be mainly referred to a reduced ventilatory capacity of the subject due to air trapping measured at rest. That could be right but it is not completely correct. The simple correlation measured by the authors does not completely clarify the reasons for the interruption of the test by patients, above or below the respiratory compensation point (RCP). Some studies had demonstrated a reduced ventilatory compensation capacity in patients with a more severe emphysema i.e Paoletti *et al*, 2011 ^1^ nerveless it should take in account that several metabolic alterations occur after the anaerobic threshold that are independent to the ventilatory mechanics and that are more closely related to physical conditioning of the patient. After the anaerobic threshold, peak ventilatory response could be related to several different causes: the exercise intensity and the power increments, the anaerobic reserve of the subject, the muscular O _2_ extraction capacity, the amount of metabolic acidosis, the increase in catecholamine levels, the developed or not of dynamic hyperinflation, the ventilatory chemiosensitivity of the carotid bodies, some cardiovascular alteration as an opening of PFO or the develop of pulmonary hypertension, even the individual motivation to complete the exercise and the subject propensity to perform that specific exercise. It is difficult to understand which is the real cause of exercise interruption and this could significantly affect the amount of nadir to peak ventilatory equivalent difference. The difference between nadir to peak ventilatory equivalents does not seems to be a good estimate of patient’s ventilatory compensation during the exercise.
2. It should be noted that, in the regression analysis there are half of the patients that did not increase ventilatory equivalents, which means that they stop the exercise at RCP or immediately after. Table 3 show a significant difference in peak RER between the patients who have not shown increments in VE/CO _2_ and the ones who have continued to increase ventilation, suggesting that the two groups reach different intensity exercise domain and probably are different populations (i.e. RC/TLC significantly different). A more complete table, that includes Watt Max, VO _2_ peak in ml/kg, VO _2 pulse_, VE/MVV _peak_ and FEV _1_/FVC of the two groups could be helpful. Unfortunately it is impossible to know why the subject who showed no increase in VE/VCO _2_, interrupt the exercise immediately after the reach of RCP. To put all the results in the same regression appear as an hazard, there are too many unpredictable sources of error. The authors must provide consistent evidence that the observed correlation is not only apparent. The estimated coefficients of the linear regression and the relative interval of confidence should be calculated. An estimation of the standard error of regression coefficient R ^2^ should be provide together with the 95% confidence band for R. A residual analysis could help to understand how good is the correlation and if the assumption of normality of the distribution is preserved.
3. The variable chosen by the authors to represent the exercise ventilatory compensation of the patients is not the one universally accepted and utilized for this propose. The authors should clarify why they have not utilized, for instance, the VE/CO _2 slope_. In a recent paper Gargiulo P. and coworkers demonstrated that the analysis of VE/CO _2 slope_ and its intercept on the abscissa (VE _int_), that represent the ventilatory requirements when pulmonary gas exchange is null, represents better the ventilatory efficiency (Gargiulo P et al. 2014 ^2^). In COPD patients VE _int_ correlate with the VD and Neder and colleagues recently showed that increases in VE _int_ better reflected the progression of functional impairment form mild to end-stage of COPD (Neder JA et al. 2015 ^3^). I suggest to test also these parameters in you analysis that are more clearly related to pulmonary efficiency and that are less dependent from the individual motivation of the patients to tolerate the effort.
4. The authors conclude the abstract suggesting that the reduced ventilatory increment in VE/VCO _2_ from rest to peak exercise could be a prognostic biomarker. This assumption is not clarified in the text, no data was provided to support this statement and the assumption remains speculative. 

Minor comments:

**Abstract**

*Line 4, p 1_* *“During incremental cardiopulmonary exercise testing, ventilatory equivalents for carbon dioxide….. may be difficult to identify in COPD patients because of limited ventilatory compensation capacity”*. This is not correct. Ventilatory equivalents are always identifiable, in COPD patients could be difficult to identify the respiratory compensation point, the point at which it could be observed an hyperventilatory response to late-exercise acidosis.

*Line 19, p 1_* *“* *D* *V _E_/VCO _2_ and* *D* *V _E_/VO _2_”*.  DV _E_/VCO _2 _abbreviation is usually utilized to indicate VE/CO _2 slope_ and could be confounding, I suggest to change it with a new acronym that specifies that it is the difference between the peak and nadir values.

*Line 28, p 1_ “…and may be candidate prognostic biomarker”* the statement is speculative and not investigated in the study.

**Introduction**

*Line 26, p 3 _* “The ventilatory threshold (VT)…. “. I suggest to utilize a different abbreviation (i.e. AT or LT), “VT” could be confused with Tidal Volume.

**Methods**

 Lines 1-10, p 4 _ *“Patients performed incremental symptom-limited cycle ergometer…”* The authors mentioned a 1-2 minute of warm-up period at 0 watt. 0 watts assumes that an ergometer with pedal assistance has been used. What kind of cycle ergometer was used? did you mean a period of free pedaling at the minimum of watts? Have you estimated the amount of the minimum load of your cycle-ergometer? 1-2 min of warm up could be correct with a real 0 watts ergometer but it could be a too short period if the minimum load provide by the ergometer was 20W or greater.

 Line 10, p4_ *“ the test was terminated at exhaustion” *Can you please better define “exhaustion”?

**Results**

*Table 1.  *The table should be integrated with the absolute value of VO _2 peak_ in ml/kg, VO _2@AT_ in ml/kg, Watt Max and RER at peak exercise. I suggest to put SpO _2 peak_  rather than the desaturation at peak effort.  An explication of how the authors calculated BR should be added to Methods. The percentage of tests in which the respiratory compensation point was identifiable should be added somewhere. DVE/CO _2_ should be change with some other abbreviation.

 Lines 5-16, p4 _ *“* *D* *V _E_/VCO _2_ (r= -0.5058, 95% CI -0,7750 to – 0.08149, p =0.0234)…”* a p = 0.0234 looks quite significantly, nerveless the adaptation of the regression appear very low, it seems that R ^2^ is not more than 0.25. I suggest to add to your plot the regression coefficients with the respective standard errors. It could be useful analyzed the residual plot in order to increase the adaptation.

 Lines 19-20, p4. *“Notably, we found no significant correlation between neither* *D* *VE/VCO _2_ nor nadir VE/VCO _2_ and peak VO _2_ or FEV _1_. "*. Do you mean the absolute values or % predicted?

*Table 3*. The table shows a significant difference in RER peak between the two population analyzed. The patients with a significant increase in *D* *VE/VCO _2_* probably achieve a greater effort intensity. As the entire correlation depends on the patients who sustain the effort beyond their VEVCO _2 nadir_ , it is likely that the correlation is no longer detectable in the more severe patients. I would like to know the differences between the two groups in VO _2 peak_ expressed as ml/kg, Watt Max, BR%, Heart Rate Reserve (220-age), VO _2 pulse_ at peak exercise, FEV _1_ in mL and FEV _1_/VC.

**Discussion**

 Lines 17-19, p 5. *“In this study we examined the ability of …. Focusing on the increase in the V _E_/VCO _2_ and V _E_/VO _2_”*. This is not correct. You have not measured any slope. You measure just a difference.

 Lines 14-19. P 6. *“Therefore the inability to compensate…”*. This statement is entirely speculative, beyond the scope of the study and not supported by any data that can be highlighted in the paper.

 Lines 28-34, p 6. *“Therefore, it seems that the lack of increment….”*  You are comparing a static measurement with a dynamic alteration that occurs in the moment of maximum stress. The problem is that you have not measured any indexes of dynamic hyperinflation. Your conclusion cannot be proven.

 Lines 34-35, p. 6 “ *however, we think that the lower respiratory exchange ratio at peak exercise  represents another manifestation of the decreased ventilatory response”.* Unfortunately it does not stand out easily if the patient has stopped due to actual exhaustion or to other reasons. Especially in a retrospective study.

 Lines 49-50, p. 6 *“Some patients were obese, which may have caused mild restriction…*” You should take in account that BMI could largely affect the VCO _2_ body storage and, as a consequence, the RCP detection or VE/VCO _2 peak_ values. Is there any differences between the two groups (No Nadir to Peak increment vs patients with nadir to peak increment) in BMI? Can you provide this data?

I confirm that I have read this submission and believe that I have an appropriate level of expertise to state that I do not consider it to be of an acceptable scientific standard, for reasons outlined above.

## References

[1] : Cardiopulmonary exercise testing (CPET) in pulmonary emphysema. *Respir Physiol Neurobiol*.2011;179(2-3) : 10.1016/j.resp.2011.07.013 167-73 10.1016/j.resp.2011.07.013 21840426

[2] : A non invasive estimate of dead space ventilation from exercise measurements. *PLoS One*.2014;9(1) : 10.1371/journal.pone.0087395 e87395 10.1371/journal.pone.0087395 24498096PMC3907547

[3] : Exercise ventilatory inefficiency in mild to end-stage COPD. *Eur Respir J*.2015;45(2) : 10.1183/09031936.00135514 377-87 10.1183/09031936.00135514 25359345

[4] : Physiological and clinical relevance of exercise ventilatory efficiency in COPD. *Eur Respir J*.49(3) : 10.1183/13993003.02036-2016 10.1183/13993003.02036-2016 28275174

[5] : Impact of chronic obstructive pulmonary disease on exercise ventilatory efficiency in heart failure. *Int J Cardiol*.2015;189: 10.1016/j.ijcard.2015.03.422 134-40 10.1016/j.ijcard.2015.03.422 25889444

# Response

**Published:** 2020-03-04

**Authors:** Samir Nusair

**Affiliations:** Clalit Health Services, Israel

The authors would like to thank Dr. Gabriele Valli for his valuable comments. This is a retrospective study with a small number of patients, and we certainly acknowledge the inherent significant limitations, which we refer to in the Discussion section. We sought to study changes in VE/VCO2 during the incremental exercise test in COPD patients, a very intriguing subject. Furthermore, we consider this work as a preliminary/exploratory study, and as that it has well acknowledged limitations and implications. Furthermore, we added data of VE/VCO2 slope and VE/VCO2 line intercept as suggested, providing further insight into our cohort. 

  

We would like to address the concerns raised by the Reviewer.

Re Major Comments:

1. We certainly agree with the reviewer that there are several possible mechanisms and factors that may contribute to exercise limitation in patients with COPD. In this study we aimed at studying the changes in VE/VCO2 during exercise in these patients. We investigated a possible relationship between airway trapping (defined by increased RV/TLC) and the decreased ability to increase ventilation in response to acidosis as seen by the lack of VE/VCO2 rise and absence of the ventilatory compensation phase seen in people without COPD. We certainly do not claim cause and effect on the basis of our findings.

  

2. We have now added information as requested by this reviewer to Table 1 and Table 3 of peak Work Rate, peak VO2 (ml/kg/min),FEV1/FVC and exercise ventilatory mechanical data (VE/MVV, peak exercise Vt/VC). 

Patients who had an increment in VE/VCO2 had higher VO2 (per body weight), higher peak WR and higher RER. Again, this is not about cause and effect but epiphenomena. Whether these patients were “more motivated” to perform exercise than those who did not cannot be absolutely excluded, yet with all patients motivated to perform exercise until exhaustion, (i.e., could not keep required cycling pace) or had marked symptoms such as shortness of breath, simple lack of motivation to exercise is unlikely sole explanation. Adding data regarding end-tidal PCO2 at baseline and at peak exercise in both groups should be also helpful. 

We sought to find a correlation between increments of ventilatory equivalents and air trapping (see Results section, paragraph 2). We did not seek to find a linear regression. We agree with the reviewer about the “hazards” in creating a linear regression of all the data, especially when many other factors could be involved. Therefore we take a step back and present the data of correlation we intended to show originally, adding it to Table 2, in the first two rows. We deleted Figure 1 as it is irrelevant.

3. In this work, we originally wanted to look at the changes in VE/VCO2 during exercise, the isocapnic buffering phase and the ventilatory compensation phase. Previous observations have noted the absence of significant ventilatory compensation in patients with COPD (Ref 6). Since there are previous descriptions of correlation between VE/VCO2 slope and air trapping (Refs 1,3 &5 in the manuscript) we attempted to somehow quantitate the increment in VE/VCO2 during incremental exercise, expecting that some patients may not have an increment (deltaVE/VCO2=0). To the best of our knowledge this is a somewhat novel approach.

In our opinion, nadir VE/VCO2 may be easier to identify in routine clinical practice and also to relate to peak VE/VCO2. In this cohort, we found a high correlation between Nadir VE/VCO2 and VE/VCO2 slope. Data of VE/VCO2 slope was added. There was a significant correlation between Nadir VE/VCO2 and VE/VCO2 slope (R=0.82 95%CI 0.59-0.92, p<0.0001).

Data of VE/VCO2 intercept was also added. (All 3 Tables). In Table 3 we show that VE/VCO2 intercept was significantly higher in patients in whom deltaVE/VCO2=0. We now show that our findings are in agreement with this parameter which represents dead space breathing.  

4. Reference to a “possible prognostic biomarker” in the end of the abstract was deleted.

 Re Minor Comments:

Abstract:

1. Sentence was corrected “During incremental cardiopulmonary exercise testing, actual nadir values of ventilatory equivalents for carbon dioxide (V _E_/VCO _2_) and oxygen (V _E_/VO _2_) may be difficult to identify….”

2. The authors have checked before writing this manuscript the use of the symbol delta, and we did not find a similar use of it in this context, even in reference to VE/VCO2 slope, therefore we resort to continue using this symbol in our manuscript. Another acronym/abbreviation will be more difficult to use at this stage.

3. The reference to “biomoarker” was omitted.

 Introduction:

1. The abbreviation “VT” in relation to ventilatory threshold was deemed unnecessary at this stage and it is no longer used.  

 Methods:

1. Sentence was re-written “Following a 1–2 minute warm-up period of unloaded exercise and with VO _2_ and VCO _2_ reaching a plateau,….”. We accept the reviewer’s note that this cannot be defined as WR=0, since it was pedaling against the inherent resistance of the cycle machinery without motored assistance.

2. Exhaustion in the context of exercise test performance is now defined. The sentence was re-written:

“The test was terminated at exhaustion, when the patient could not keep the required cycling pace or asked for exercise termination.”

 Results:

1. More information added to Table 1 and other tables. Breathing reserve is defined at the bottom of Table 1.

The percentage of patients with identified respiratory compensation point would be 50% and is represented by a deltaVE/VCO2>0.

2. As noted above we performed correlation estimations and not linear regression (explained above and changes described)

3. There was no correlation between neither deltaVE/VCO2 nor nadir VE/VCO2 and peak VO2 and FEV1, as percentage of predicted. We added also a correlation with VO2 in ml/kg/min, which just achieved a significant correlation with a p=0.0462.  

4. Table 3 is now expanded and more parameters are compared.

 Discussion:

1. The sentence now has been changed “…..focusing on the increase in V _E_/VCO _2_ and V _E_/VO _2_ that occur following the ventilatory compensation point.” The word “slopes” was deleted.

2. The entire sentence “Therefore, inability to compensate for the increased ventilatory demands of acidosis may contribute to termination of exercise at lower exercise intensity.” was deleted.

3. The sentence has been changed and “can be related to” is changed to “is correlated with”. 

4. Instead of “represents” the phrase is changed to “may represent”, thus expressing some reservation about this conclusion.

5. Comparison of BMI between the two groups has been added. There was no difference in BMI between the two groups.
